# Effect of Ras Inhibition in Hematopoiesis and BCR/ABL Leukemogenesis

**DOI:** 10.1186/1756-8722-1-5

**Published:** 2008-06-05

**Authors:** Karina J Baum, Ruibao Ren

**Affiliations:** 1 Rosenstiel Basic Medical Sciences Research Center, Department of Biology, Brandeis University, Waltham, MA 02454, USA; 2 Current address : College of General Studies, Boston University, 871 Commonwealth Avenue, Boston, MA 02215, USA

## Abstract

Ras small GTPases are activated in many hematopoietic growth factor signaling and in hematological malignancies, but their role in hematopoiesis and leukemogenesis is not completely known. Here we examined the effect of Ras inhibition by a dominant negative mutant of Ras, N17 H-Ras, in adult hematopoiesis and in BCR/ABL leukemogenesis using the mouse bone marrow transduction and transplantation approach. We found that N17 H-Ras expression suppressed B- and T-lymphopoiesis and erythropoiesis. Interestingly, N17 H-Ras did not suppress myelopoiesis in the bone marrow, yet it greatly attenuated BCR/ABL-induced chronic myelogenous leukemia (CML)-like myeloproliferative disease. Most BCR/ABL + N17 H-Ras mice eventually developed pro-B lymphoblastic leukemia/lymphoma (B-ALL). These results suggest that Ras activation is essential for the development of lymphoid and erythroid cells but not myeloid cells and that Ras is a critical target of BCR/ABL in the pathogenesis of CML, but not B-ALL.

## Background

Ras proteins are small GTPases that act as molecular switches, transducing signals from activated receptors to downstream effectors to regulate cell proliferation, survival and differentiation [1]. Members of the Ras family include three cellular *Ras *genes, which encode four highly homologous proteins: H-, N-, and K-Ras4A and 4B, the latter two being alternatively spliced isoforms differing only at the carboxyl terminus (with alternative 4^th ^exon) [2]. H-, N-, and K-Ras proteins are widely expressed, with K-Ras expressing in almost all cell types [3]. The four Ras proteins share an identical amino-terminal region. This region includes the nucleotide binding and effector loop domains, providing a basis for the ability of Ras proteins to interact with a common set of activators and effectors and to share many biochemical and biological functions. The next 80 amino acids are also highly homologous (85% identity) among all four Ras proteins. Conversely, the Ras proteins diverge highly at the carboxyl-terminal 23–24 amino acids, referred to as the hypervariable domain (HVR) [4]. This HVR domain signals the post-translational modifications that enable the Ras proteins to attach to the inner surface of the plasma membrane, a prerequisite for Ras-mediated signal transduction. Different modifications and targeting mechanisms result in localization of the Ras proteins to functionally distinct microdomains of the plasma membrane, which may allow the Ras proteins access to different pools of Ras regulators and/or effectors and, therefore, generate distinct signal outputs [3].

Gene knockout studies in mice have revealed functional differences as well as redundancies between Ras proteins. Mice that lack the expression of N-Ras or H-Ras or both are viable and have no obvious abnormal phenotype [5,6]. But the mice lacking the *K-Ras *gene die during embryonic development between days 12 (E12) and 14 (E14), with fetal liver defects and evidence of anemia [7,8]. These results demonstrate that K-Ras provides a unique and essential function during mouse development. Interestingly, the mice heterozygous for K-Ras (K-Ras^+/-^) and homozygous null for N-Ras (N-Ras^-/-^) die between E10 and 12, while the K-Ras^+/- ^mice are normal. Furthermore, no viable double homozygous mutant (K-Ras^-/-^; N-Ras^-/-^) was recovered at E9.5. These results suggest that there is partial redundancy between Ras proteins [7].

In addition to their normal cellular functions, Ras proteins play critical roles in tumorigenesis. Mutated *Ras *genes are associated with approximately 30% of all human cancers, including both solid tumors and hematological malignancies [9]. In addition to the direct activation by mutations, Ras can also be functionally activated by other oncogenic mutations, such as the BCR/ABL fusion protein.

*BCR/ABL *is produced when the breakpoint cluster region gene (*BCR*) sequences on chromosome 22 are fused to *ABL *sequences on chromosome 9 by a reciprocal translocation [10]. The BCR/ABL fusion protein is present in nearly all patients with chronic myelogenous leukemia (CML) and in 20% of the adult and 2–5% of the pediatric patients with B-acute lymphoblastic leukemia (B-ALL). We and others have shown that expression of BCR/ABL in mouse bone marrow cells by retroviral transduction efficiently induces a myeloproliferative disease (MPD) resembling human CML [11,12]. In this model system, BCR-ABL with a mutation (Y177F) at the tyrosine-177 residue – a high affinity-binding site for the Grb2 SH2 domain when phosphorylated [13,14] – induced a T cell leukemia and lymphoma after a prolonged latent period [15-17]. Grb2 is an SH2- and SH3-containing adapter protein that binds Sos – a guanine nucleotide exchange factor (GEF) of Ras – as well as the scaffolding adapter Gab2 [18]. These interactions activate Ras, and recruits phosphotidylinositol-3 kinase (PI3-K) and the protein tyrosine phosphatase SHP2 [13,14,18]. The importance of Y177 in the induction of CML-like MPD by BCR-ABL suggests that activation of Ras signaling pathways plays a critical role in the pathogenesis of CML. Consistent with this result, BCR/ABL transformation was shown to be blocked by interfering with Ras function using either a dominant negative mutant of Ras or the catalytic domain of Ras-GAP [19].

The redundant function among Ras proteins makes it difficult to assess the importance of Ras in development and tumorigenesis. The Ras mutant that contains an asparagine at position 17 functions as a competitive inhibitor of the normal endogenous wild-type Ras for the binding of GEFs [1]. The N17 H-Ras mutant has been shown to be the most effective in inhibiting the activation of all three isoforms of Ras in the cell, due to the wide distribution of this protein throughout the plasma membrane as compared to K-Ras and N-Ras [20-22]. To assess the role of Ras in adult hematopoiesis and in BCR/ABL leukemogenesis, and to assess the importance of Ras as a therapeutic target, we examined the effect of N17 H-Ras mutant in bone marrow reconstitution and in the induction of CML-like MPD by BCR/ABL in mice.

## Methods

### DNA constructs

A vector capable of expressing N17 H-Ras and BCR/ABL fused to GFP on the 3' end of BCR/ABL in identical levels was made through a multistep cloning strategy. The MSCV-BCR/ABL-GFP-IRES-N17Ras was constructed as follows: The dominant negative Ras (N17 H-Ras) (generous gift provided by Dr. Hong Cai in Dana-Farber Cancer Institute) was amplified by PCR using the primers 5'CGC GGA TCC ATG ACA GAA TAC AAG CTT GTG GTC3' and 5'ACG CGT CGA CTC AGG AGA GCA CAC ACT TGC AGC T3' which contain a Bam HI and Sal I internal sites. The PCR product was digested with Bam HI and Sal I, cloned into pBluescript II/SK^- ^(Stratagene, La Jolla, CA) into the Bam HI/Sal I sites, and then sequenced to verify that there were no introduced mutations. This N17 H-Ras sequence was then compared with the H-Ras sequence of the Genbank database (Accession #J00277). To clone the N17 H-Ras fragment into the pCITE (Novagen, Madison, WI) vector that carries the IRES (internal ribosomal entry site) sequence necessary to allow the Ras expression, the N17 H-Ras fragment was ligated to the annealed adapters 5' CAT GGC GGC CGC TG 3' and 3' CGC CGG CGA CCT AG 5' to generate a Nco I site which was necessary for cloning into pCITE. This construct was then cut with Eco RI and Sal I to isolate the Ras insert to clone it into MSCV retroviral vector [23]. This vector is capable of transducing genes with high efficiency into undifferentiated murine embryonic carcinomas and hematopoietic stem cells. GFP or BCR/ABL-GFP fusion protein was cloned into the EcoRI site of MSCV as described previously [24]. In order to clone the IRES-neo fragment a similar strategy as described above was followed to clone the other construct MSCV-BCR/ABL-GFP-IRES-Neo. Another vector capable of expressing N17 H-Ras and GFP on the 5': MSCV-GFP-IRES-N17 H-Ras was constructed as follows: The GFP was amplified by PCR using the primers 5' AAG AAT TCG CGG CCG CCA TGA CAG AAT ACA AGC TTG TGG TGG TG3' AND 5'AAG AAT TCG CGG CCG CTC AGG AGA GCA CAC ATT GCA GCT CAT G3' which contain an Eco RI and Not I internal sites. The PCR product was digested with Eco RI, cloned into MSCV-IRES-N17 H-Ras into the Eco RI site. Orientation was checked by enzyme digestion. Finally, the MSCV-IRES-GFP vector control was constructed as described before [12].

### Cell culture, retrovirus preparation and viral titer quantification

NIH 3T3 and BOSC 23 cells were grown as previously described [25,26]. Helper-free retroviruses were generated by transient transfection of retroviral DNA constructs into BOSC 23 cells as previously described [12,25]. Retroviral transduction and tittering was performed as described [12,25]. All viruses were adjusted to equal titer based on GFP expression just before transduction of BM cells or other cell types.

### Transformation assays of cell lines in vitro

NIH 3T3 cells (1 × 10^5^) were plated on 60 mm Petri dish. After 18 hours the cells were infected for 4 hs with 1 ml, 0.5 ml, 0.1 ml, 0.01 ml, or 0.001 ml of retroviral supernatant per duplicate plates. Seven to ten days after infections the plates corresponding to the different retroviral dilutions were analyzed and the number of Foci quantified. For bone marrow soft agar colony assays, the retroviral-transduced cells were washed twice in excess phosphate-buffered saline (PBS) (GIBCO BRL, Grand Island, N.Y.), counted and plated into six-well plates at 10^5 ^cells per well (0.3% Bacto agar, 20% FBS, 200 U of penicillin/ml, 200 μg of streptomycin/ml, 50 M 2-mercaptoethanol in DMEM).

### Infection, transplantation and colony assay of bone marrow cells

Bone marrow cell infection and transplantation were performed as described previously [12]. Briefly, 5-fluoroucil (5-FU)- treated bone marrow cells (1 × 10^6 ^cells/ml) from male Balb/c (Taconic Farm, Germantown, NY) donor mice (5 to 6 weeks old) were infected for 24 hours in a cocktail containing DMEM, 15% FCS, 5% WEHI-conditioned medium, 7 ng/ml IL-3 (Genzyme, Cambridge, MA), 12 ng/ml IL-6 (R&D System, Minneapolis, MN), 56 ng/ml stem cell factor (SCF, R&D System), 2 mM L-glutamine (GIBCO BRL), 100 μg/ml streptomycin, 100 U/ml penicillin, 0.25 μg/ml amphotericin B (GIBCO BRL), 2 μg/ml polybrene and 30% of retroviral supernatant. After 24 hours, the bone marrow cells were re-infected with freshly made retroviruses. Two days post infection, the bone marrow cells were washed once with PBS and then injected into lethally irradiated (2 doses of 450 rads given 4 hours apart) syngeneic female BALB/c mice (5 to 6 weeks old) through tail vein injection of 4 × 10^5 ^cells per mouse. The secondary bone marrow transplant was performed with 5 × 10^6 ^whole bone marrow cells from mice reconstituted with MSCV-IRES-GFP or MSCV-GFP-IRES-N17 Ras bone marrow cells into lethaly irradiated BALB/c mice.

In vitro soft agar colony assays were performed as described previously [26] with modifications. From the infected cells used for bone marrow transplantation, 10^5 ^infected BM cells were plated in 35-mm-diameter cells in DMEM, 20% FCS, 100 μg of streptomycin/ml, 100 U of penicillin/ml, 0.25 μg of amphoterecin B/ml, 50 μM 2-mercaptoethanol (Sigma), and 0.3% bacto-agar, on top of a layer of medium containing 0.6% bacto-agar. Colonies were counted on day 15. BM liquid cultures were grown in the absence of cytokines. Cytospin preparations of these cultures were stained with Hema-3.

### In vitro methylcellulose colony assays

Bone marrow cells for *in vitro *methylcellulose colony assays were either isolated from bone marrow of our retroviral transduced mice or were obtained from double retroviral infection of 5-FU enriched isolated bone marrow cells from 5–6 weeks male Balb/c mice (Taconic Farm, Germantown, NY) [12]. Upon isolation, these cells were immediately sorted for GFP positive populations as described previously [12,25].

MethoCult™ CF M3434 and MethoCult™ M3334 (StemCell Technologies, Vancouver, BC, Canada) were used for myeloid and erythroid colony assays respectively. Sorted GFP positive cells were diluted to 2.0 × 10^5 ^cells/mL in Isccove's MDM (IMDM, StemCell Technologies, Inc.) with 2% FBS for MethoCult™ CF M3434 and 2 × 10^6 ^cells/mL for MethoCult™ M3334. 0.3 mL of cells were added to aliquoted 3 mL of MethoCult™ CF M3434 and MethoCult™ M3334 for duplicate cultures. Tubes of the MethoCult™ CF M3434 and MethoCult™ M3334 containing the cells were vortexed and bubbles were allowed to rise and dissipate for 5–10 minutes. 1.1 mL of the culture was dispensed into each of two 35 mm culture dishes using a 3 cc syringe and 16 g blunt end needle. Cultures in MethoCult™ CF M3434 were incubated for 10 days and cultures in MethoCult™ M3334 for 5 days at 37°C and 5% CO_2_a humidified incubator.

### Analysis of disease phenotype in mice

Hematopathological analysis was performed as previously described [12,25]. Total blood cells and white blood cells (WBCs) were counted on Coulter Counter (Model Z1; Coulter Particle Characterization, Hialeah, Fla.). Immunophenotypes of hematopoietic cells and leukemic cells were determined by flow cytometry. Flow cytometry and cell sorting were performed as described [12].

### Western blot analysis

Total cell lysates of BM cells or NIH 3T3 cells were prepared as described [25]. Proteins were separated on a 6–15% gradient SDS-polyacrilamide gel electrophoresis (SDS-PAGE) and electrophoretically transferred to nitrocellulose filters (Schleicher & Schuell, Keene, NH). The filters were probed with anti-Abl monoclonal antibody Ab-3 (Oncogene Research Products, Cambridge, MA), rat anti-Ras monoclonal antibody (Santa Cruz Biotechnology, Inc. Santa Cruz, CA), anti-dynamin (Transduction Laboratories, Lexington, KY), anti-actin (clone AC-40, Sigma, St. Louis, MO, USA), anti-phospho-p44/42 (Thr202/Tyr204) MAP kinases (Erk1 and Erk2), anti-p44/42 MAP kinases (Erk1 and Erk2) (New England Biolabs, Beverly, MA, USA). Bound antibodies were visualized by using horseradish peroxidase-conjugated anti-mouse, anti rabbit, or anti-rat IgG respectively (Southern Biotechnology Associates, Inc., Birmingham, AL, USA).

### Mice perfusion

Mice were anesthetized and then cut from the lower abdomen to the chest to expose heart, lungs, liver and thymus. A 18-gauge needle was inserted through the left ventricle of the heart into the ascending aorta, and slowly perfused the animal with 50 ml room temperature PBS until the blood was replaced by the saline fluid and liver was blanched to a yellow color. The right atrium of the heart was cut to suction the outflow.

## Results

### Expression of N17 H-Ras inhibits hematopoiesis

Prior to examine the role of Ras activation in BCR/ABL leukemogenesis using N17 H-Ras, we examined the effect of N17 H-Ras in hematopoiesis. To facilitate identification of N17 H-Ras expressing cells and determination of virus titers, we cloned into MSCV the gene encoding GFP linked together with the *N17 H-Ras *mutant gene by an internal ribosome entry site (IRES), which allows both GFP and N17 H-Ras to be translated from the same transcript. The MSCV-IRES-GFP retroviral vector was used as a control. The expression of N17 H-Ras was confirmed in infected NIH 3T3 cells (data not shown).

**Figure 1:**
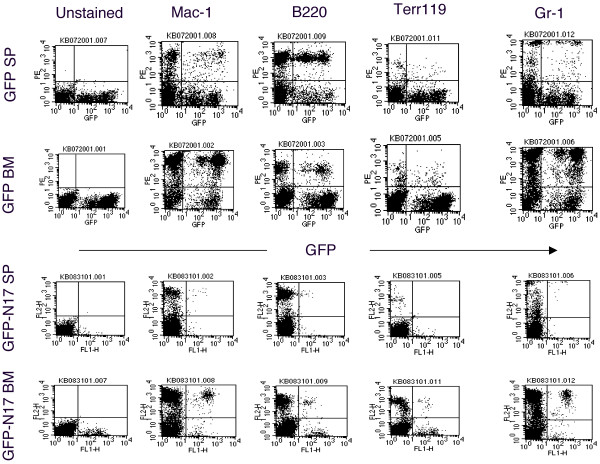
**Flow cytometric analysis of spleen and bone marrow cells from N17 H-Ras and GFP control mice**. Expression of GFP (X-axis) and Mac-1, B220, Terr-119, or Gr-1 (Y-axis) in bone marrow cells and splenocytes isolated from N17 H-Ras and GFP control mice was analyzed by flow cytometry. Results are representative of one of the three independent experiments.

To introduce N17 H-Ras into hematopoietic stem/progenitor cells, we infected the 5-FU-treated primary bone marrow cells with N17 H-Ras or GFP control retroviruses and then transplanted the infected cells to recipient mice (see Material and Methods). Four weeks after bone marrow transplantation (BMT), GFP-positive cells were seen in the bone marrow, spleen, and peripheral white blood cells of mice that received the control vector (Figure 1 and data not shown). GFP-positive cells were greatly reduced in mice that were transplanted with bone marrow cells infected with the N17 H-Ras construct (Figure 1). Similar results were seen in the bone marrow of N17 H-Ras mice 8 and 12 weeks post-BMT (data not shown). These results indicate that suppression of Ras activation by N17 H-Ras inhibits bone marrow reconstitution/hematopoiesis. The effect of N17 H-Ras in the development of specific blood lineages was analyzed as follows.

### Ras activation is critical for B-lymphopoiesis

B220^+ ^cells with various levels of GFP expression (both high and low) were seen in GFP control mice (Figure 1). The number of GFP-positive B220^+ ^cells was much lower in mice that were transplanted with bone marrow cells infected with the N17 H-Ras construct than that in GFP control mice (Figure 1). In particular, GFP^high ^B220^+ ^cells were not detected in N17 H-Ras mice. Retrovirus-mediated transgene expression varies in target cells depending on its site of integration in the host chromosome. It has been shown that there is a tight correlation of the expression level of the transgene placed downstream of the IRES and the expression of the transgene cloned between the retroviral LTR and the IRES [27]. The absence of B220^+ ^cells expressing high levels of GFP in N17 H-Ras mice suggests that high expression of N17 H-Ras, thus more effective inhibition of Ras, suppresses B-lymphopoiesis. In other words, certain level of Ras activation is essential for B-lymphopoiesis. We further examined the phenotype of the remaining B220^+ ^cells (GFP^low^).

**Figure 2:**
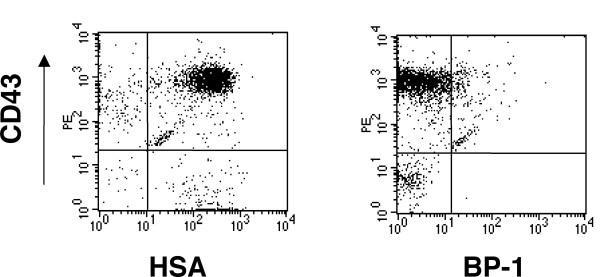
**Immunophenotyping of GFP^low ^B-lymphoid cells from bone marrow of N17 H-Ras mice**. Flow cytometry analysis of gated GFP-positive B220^+ ^bone marrow cells isolated from N17 H-Ras mice. Cells were stained with CD43, HAS and BP-1 as indicated.

In mouse bone marrow, all B lineage cells express in their surface the B220 (CD45R) marker. In addition, B lymphoid cells can be identified as seven distinct categories corresponding to specific stages of differentiation by analyzing the expression of CD43, HSA (CD24), BP-1, IgM and IgD [28]. We found that GFP and B220 gated bone marrow cells from the N17 Ras transduced mice were CD43^+^, HSA^+^, BP-1^- ^and IgM^- ^(Figure 2 and data not shown). These results indicate that B cell development can be blocked at the pro-B cell stage even with low levels of N17 H-Ras expression. In other words, the development of B-lymphocytes from pro-B to pre-B cell stage requires high levels of Ras activation.

### Ras activation is required for erythropoiesis

Like B lymphoid cells, the number of GFP-positive Ter119^+ ^(erythroid) cells was also significantly reduced in N17 H-Ras mice compared to that in GFP control mice (Figure 1). GFP^high ^erythroid cells were absent in N17 H-Ras mice, too. These results suggest that Ras activation is required for erythropoiesis.

**Figure 3:**
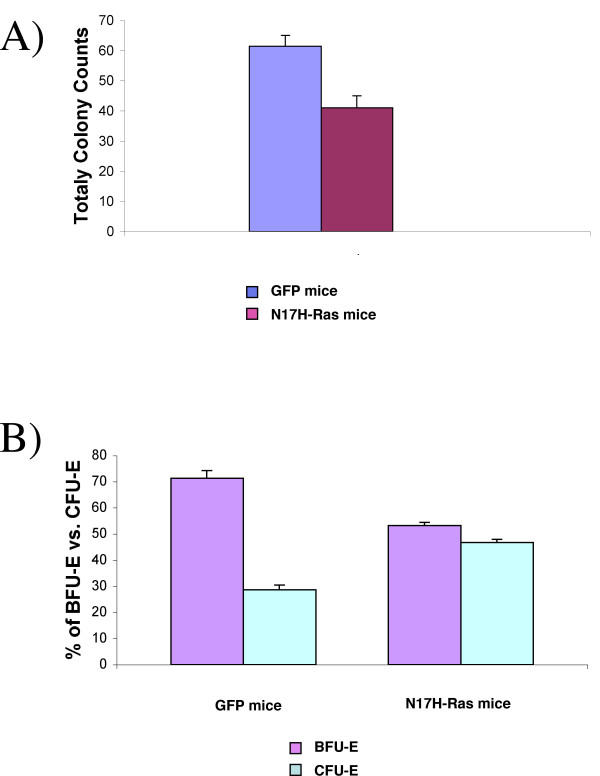
**Ras inhibition affects erythroid proliferation and differentiation *in vitro***. A). Total colony counts of sorted GFP bone marrow cells from N17 Ras and control GFP mice. The same number of cells was plated in methylcellulose media in the presence of Epo. Colonies were counted 2 and 5 days after plating for BFU-Es and CFU-Es respectively. Results revealed that the growth of bone marrow cells is affected by Ras inhibition. B) The number of BFU-E and CFU-E colonies derived from GFP sorted cells from N17 Ras and control GFP mice were quantified as the percentage of total number of erythroid colonies present for each group at day 5. Results are shown as the mean ± standard deviation of two independent experiments.

To evaluate response of the remaining GFP^low ^erythroid cells to erythropoietin (Epo), we isolated GFP positive bone marrow cells from N17 H-Ras mice and control mice by FACS sorting and then plated the same number of cells on methylcellulose in the presence of Epo. The total number of erythroid colonies was significantly decreased under Ras inhibition (Figure 3A). The decrease is largely attributed to BFU-Es, since the ratio of CFU-E to BFU-E was much higher for the N17 H-Ras than the GFP control (Figure 3B). This result indicates that full Ras activation is required for efficient growth of BFU-E in response to Epo.

### Ras activation is essential for thymocyte development

**Figure 4:**
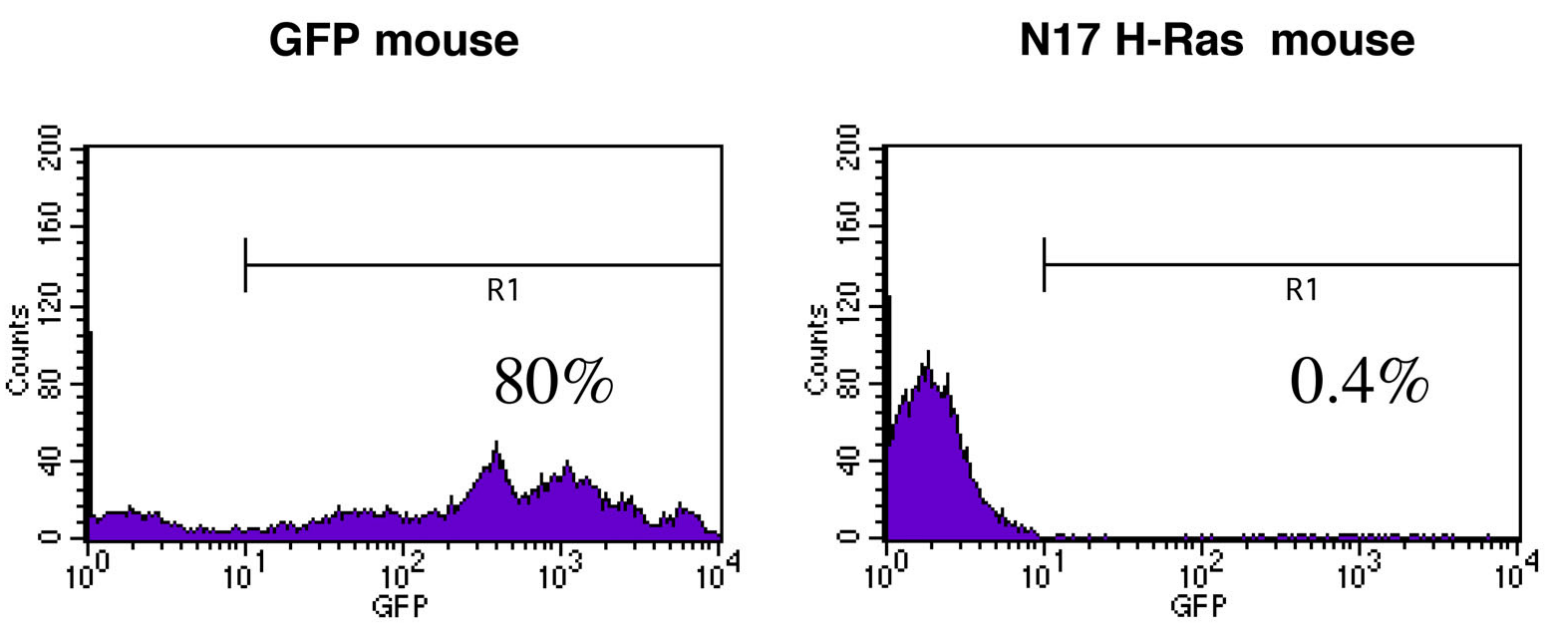
**Ras activation is essential for thymocyte development**. Thymocytes from control and N17 Ras mice were analyzed by flow cytometry after whole mouse perfusion for the detection of GFP cells.

To assess the role of Ras in thymocyte development, we examined whole thymuses of N17 H-Ras and GFP control mice for the presence of GFP positive cells. To prevent contamination of GFP positive cells present in the circulation of these mice, we perfused the mice with PBS before isolating the thymuses from the control and N17 H-Ras mice. Flow cytometric analyses of the control mice show the presence of GFP positive T cells in both the thymus and periphery (Figure 4 and data not shown). However, the absence of GFP positive cells in the periphery of the N17 Ras mice, together with the very low percentage (~0.4%) of GFP positive cells in the thymuses of these mice (Figure 4 and data not shown) indicates that inhibition of Ras activation blocks thymocyte development. The presence of some GFP positive cells in the thymus suggests that the block is not at the level of migration of lymphoid progenitor cells from bone marrow to thymus.

### Ras activation is not required for myeloid cell development in the bone marrow

Even though constitutive activation and mutations in the Ras genes have been directly implicated in many forms of malignancies of myeloid hematological origin [9], Ras role in normal myeloid lineage development was not known. To better understand Ras function in myelopoiesis we analyzed the presence of myeloid cells by staining Mac-1 and Gr-1 markers in N17 H-Ras and GFP control. Surprisingly, a relatively large population of GFP^+ ^Mac-1/Gr-1 positive cells was seen in the bone marrow (Figure 1). In particular, unlike B lymphoid and erythroid cells, there are both GFP^high ^and GFP^low ^myeloid cell populations. Western blot analysis shows that N17 H-Ras is still expressed in bone marrow cells (data not shown).

**Figure 5:**
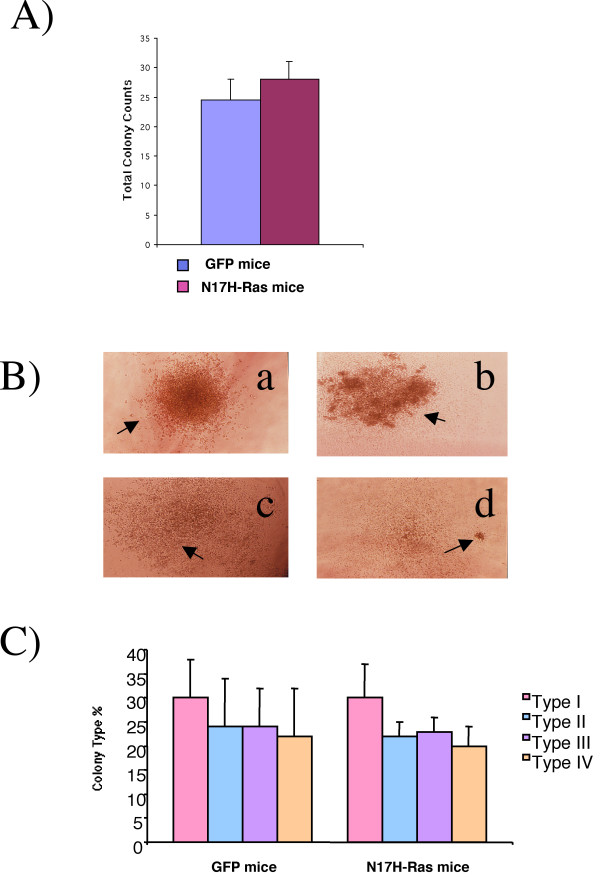
**Ras inhibition does not affect myeloid proliferation and differentiation *in vitro***. A). Total colony counts of sorted GFP bone marrow cells from N17 Ras and control GFP mice. The same number of cells was plated in methylcellulose media in the presence of IL-3, IL-6 and SCF. Colonies were counted 10 days after plating. B) Colonies derived from GFP sorted cells from N17 Ras and control GFP mice were morphologically characterized 10 days after plating. Four different kinds of CFU-GM colonies: Type a (I), b (II), c (III), and d (IV) were identified for both N17 Ras and GFP control mice. C) Colonies were quantified as the percentage of each of the types present for both groups. Results are shown as the mean ± standard deviation of three independent experiments.

To further evaluate the role of Ras activation in myeloid cell proliferation and differentiation, we examined the effect of N17 H-Ras in bone marrow cell growth in vitro. Bone marrow cells from N17 H-Ras mice and control mice were sorted for GFP positive populations and then the same number of cells were plated and cultured on methylcellulose in the presence of cytokines that support myeloid cell growth. We found that the total number of myeloid colonies formed from bone marrow cells of N17 H-Ras mice and of control mice were similar (Figure 5). Moreover, differentiation of myeloid cells was not affected by Ras inhibition as seen by the growth and differentiation of those cells into different types of CFU-GM colonies (Type I, II, III, and IV) (Figure 5). Together, these results suggest that Ras activation seems to be dispensable in the development of myeloid cells in the bone marrow. The lower total number of GFP positive myeloid cells seen in N17 H-Ras mice (Figure 1) may be due to the reduction of more primitive progenitor cells.

### Ras activation is required for the induction of CML-like MPD by BCR/ABL

**Figure 6:**
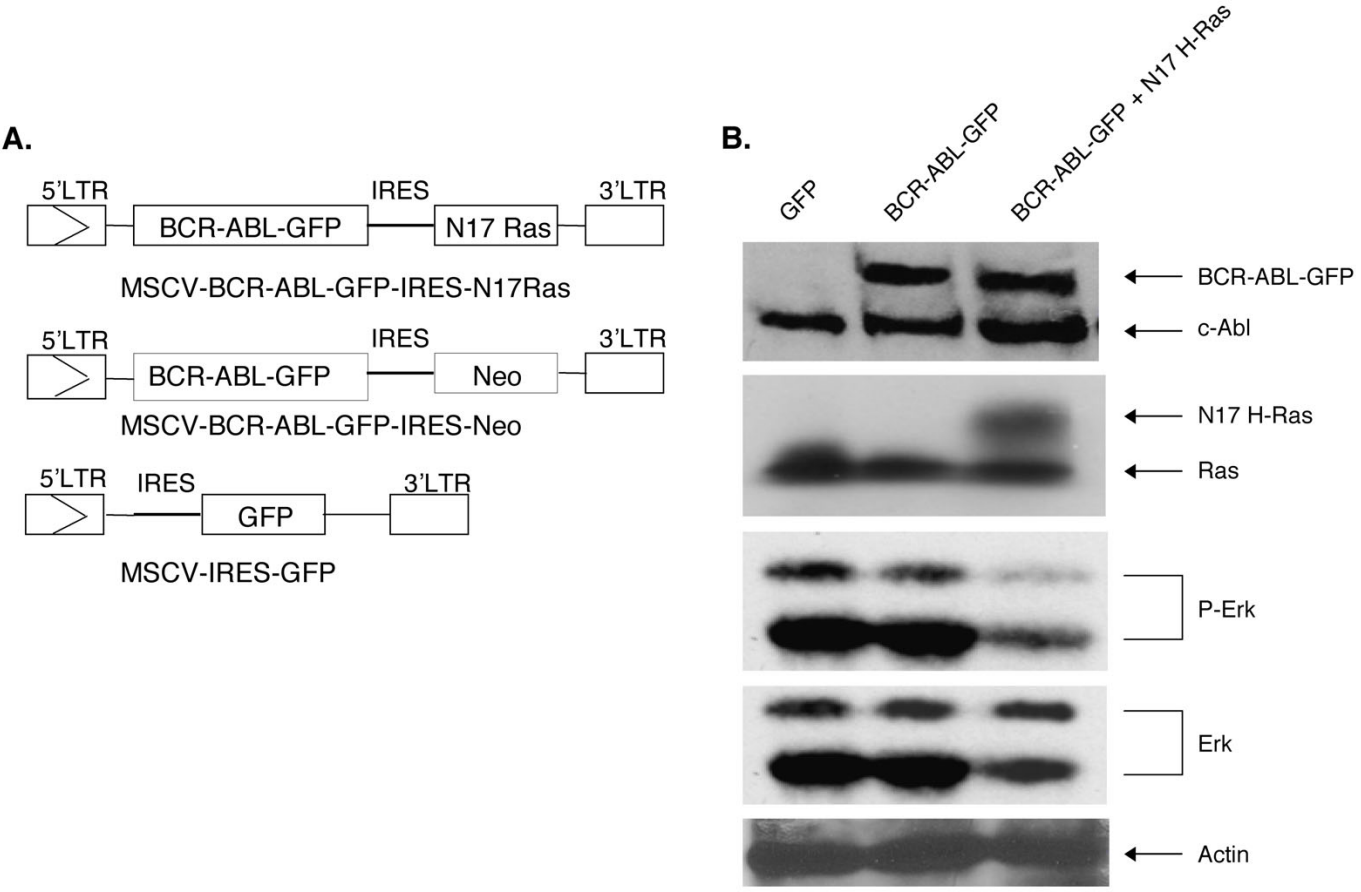
**Structure of retroviral vectors used to transduce GFP, BCR/ABL-GFP and BCR/ABL-GFP + N17 H-Ras and their expression in NIH 3T3 cells**. (A) LTR: long terminal repeat. Enzyme abbreviations: Cl, *Cla *I; RI, *EcoR*I; N, *Not *I; S, *Sal *I. (B) NIH 3T3 cells were infected with titer-matched MSCV-IRES-GFP (lane 1), MSCV-BCR/ABL-GFP-IRES-Neo (lane 2), and MSCV- BCR/ABL-GFP-IRES-N17 H-Ras (lane 3) retroviruses. The corresponding cell lysates were subjected to SDS-PAGE and analyzed by Western blot, probed with anti-Abl monoclonal antibody AB-3, anti-Ras monoclonal antibody and anti-dynamin (as a loading control).

Having shown that myelopoiesis is not suppressed by expression of N17 H-Ras, we moved to examine the effect of N17 H-Ras in BCR/ABL leukemogenesis. We generated MSCV-BCR/ABL-GFP-IRES-N17H-Ras and MSCV-BCR/ABL-GFP-IRES-Neo retroviral constructs (Figure 6A). Fusing GFP to BCR/ABL facilitates viral tittering and identification of infected cells. We have shown previously that BCR/ABL-GFP still induces CML-like MPD efficiently [12]. A neomycin gene (Neo) was cloned downstream of IRES as a control. To characterize these constructs, we infected NIH 3T3 cells with MSCV-BCR/ABL-GFP-IRES-N17H-Ras, MSCV-BCR/ABL-GFP-IRES-Neo, and MSCV-IRES-GFP retroviruses, respectively. Figure 6B shows that infection of NIH 3T3 cells with titer-matched MSCV-BCR/ABL-GFP-IRES-N17H-Ras and MSCV-BCR/ABL-GFP-IRES-Neo retroviruses yielded similar levels of BCR/ABL-GFP expression, as shown by western blotting using an anti-Abl antibody. The expression of N17 H-Ras appears as a bigger protein compared to the endogenous Ras (lane 3). This difference in size is due to the presence of an adapter introduced to the N-terminus of the protein (see Materials and Methods). Consistent with the inhibition of Ras activation by N17 H-Ras, phosphorylation of Erk1 and 2 proteins was reduced in NIH 3T3 cells expressing BCR/ABL-GFP and N17H-Ras compared to that in NIH 3T3 cells expressing BCR/ABL-GFP and Neo (Figure 6B).

To characterize the transforming potential of MSCV-BCR/ABL-GFP-IRES-N17H-Ras and MSCV-BCR/ABL-GFP-IRES-Neo constructs, we performed *in vitro *transformation assays using NIH 3T3 fibroblasts and primary BM cells. As shown previously [19], expression of N17 H-Ras greatly inhibited BCR/ABL transformation in all these cells (Table 1).

**Table 1:** Transforming potential of BCR-ABL-GFP vs. BCR-ABL-GFP + N17 H-Ras

Cells	Assays	BCR-ABL-GFP*	BCR-ABL-GFP + N17 H-Ras*	GFP vector*
NIH 3T3	# of foci	117 +/- 16†	11 +/- 2	-
Bone marrow	# of colonies	16 +/- 0.6	0.33 +/- 0.33	-

* Retroviral titers were matched.

†Results are shown as the mean ± SD of three independent experiments.

**Figure 7:**
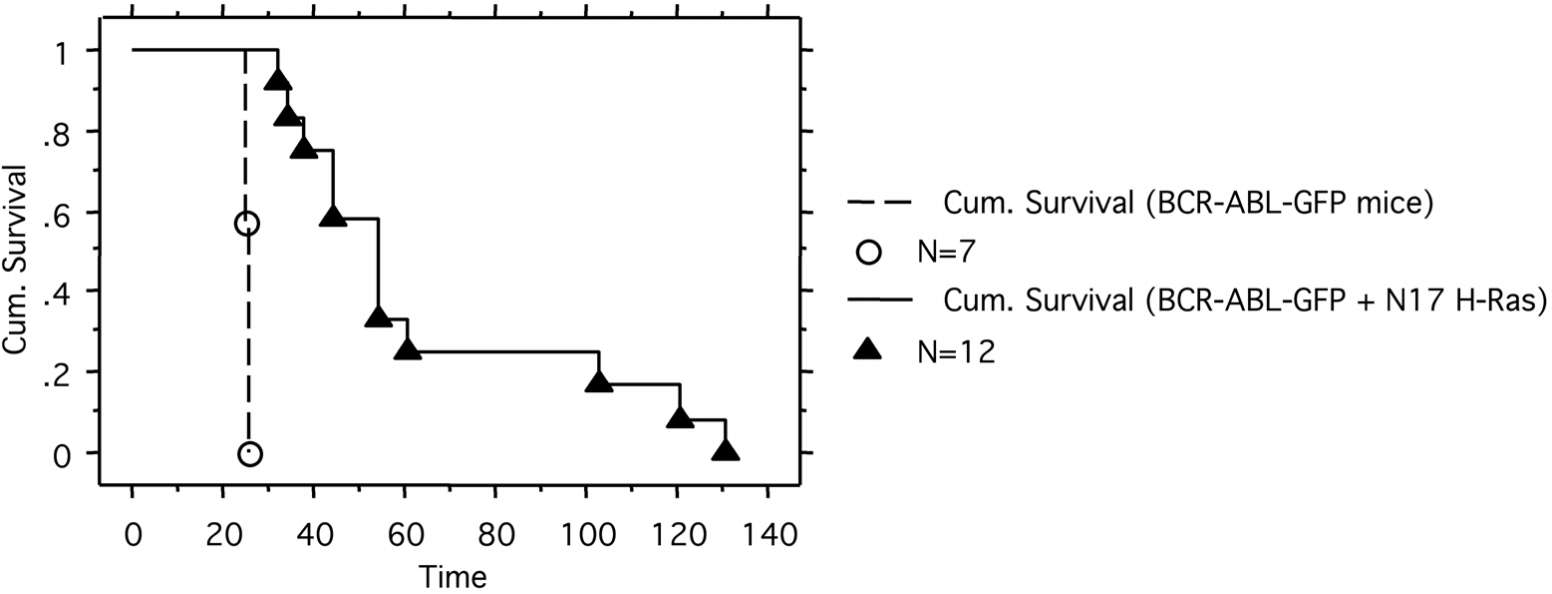
**Cumulative survival of BCR/ABL-GFP and BCR/ABL-GFP + N17 H-Ras mice**. Survival curve of BCR/ABL-GFP and BCR/ABL-GFP + N17 H-Ras mice was generated by Kaplan-Meier survival analysis. N: number of recipient mice.

To examine the effect of N17 H-Ras in BCR/ABL leukemogenesis in vivo, we transplanted the MSCV-BCR/ABL-GFP-IRES-N17H-Ras, MSCV-BCR/ABL-GFP-IRES-Neo, or MSCV-IRES-GFP-infected 5-FU treated bone marrow cells into lethally irradiated syngeneic recipient mice. As expected, BCR/ABL-GFP rapidly induced a fatal CML-like MPD (Figure 7 and data not shown). The BCR/ABL-GFP + N17Ras mice also developed a fatal disease, but with a much longer latent period compared to BCR/ABL-GFP mice (Figure 7). No sign of disease was found in GFP control mice in the same period of time (data not shown).

As shown previously, BCR/ABL-GFP induced murine CML-like MPD is characterized by high white blood cell (WBC) counts with granulocyte predominance, hepatosplenomegaly and pulmonary hemorrhages owing to extensive granulocyte infiltration and extramedullary hematopoiesis (data not shown). In contrast, myeloproliferation was moderate in BCR/ABL-GFP + N17 H-Ras mice (data not shown). More importantly the myeloproliferation disorder was transient. The vast majority of BCR/ABL-GFP + N17 H-Ras mice contain increasing amount of pro-B cells and eventually succumbed to B lymphoblastic leukemia and/or lymphoma (Table 2 and data not shown). These results suggest that Ras activation plays a critical role in BCR/ABL-induced MPD but not lymphoid malignancy.

**Table 2:** Summary of diseases developed in BCR-ABL-GFP and BCR-ABL-GFP + N17 H-Ras mice

Mice	CML	B-lymphoid leukemia/lymphoma	ND*
BCR-ABL-GFP	7/7	0/7	-
BCR-ABL-GFP+ N17 H-Ras	2/12	8/12	2/12

* Not determined.

## Discussion

Expression of the *N17 H-Ras *transgene under the control of the Eμ enhancer and the *lck *proximal promoter arrests B cell development at the pro-B cell stage [29]. Our experiment did not show GFP^high ^B lymphoid cells, suggesting that high-levels of N17 H-Ras expression completely suppressed B-lymphopoiesis. There are two critical differences between our experiment and the previous one. One difference is the cells into which the N17 H-Ras was targeted. In previous experiment, the Eμ enhancer first becomes active in the pre-pro-B cells, whereas in our experiment, MSCV may drive the transgene expression in more primitive progenitor cells and completely block differentiation of such cells into B cell lineage. The other essential difference involves the expression levels of N17 H-Ras. Retrovirus-mediated transgene expression varies in target cells depending on its site of integration in the host chromosome. When a strong retroviral LTR promoter/enhancer is combined with certain integration sites, the transgene can be highly expressed in target cells. This may result in more effective suppression of Ras activation. Our finding that low expression of N17 H-Ras blocked B-lymphocyte development at the pro-B cell stage is consonant with, and builds upon, what was found previously. Taken together, these results offer two insights. First, certain levels of Ras activation are required for the earliest B-lymphocyte development. Second, the development of B-lymphocytes from pro-B to pre-B cell stage requires high levels of Ras activation.

Expression of the *N17 H-Ras *transgene under the control of the *lck *proximal promoter has been shown to nearly completely block the proliferation of thymocytes in response to T cell receptor (TCR) stimulation and severely impair the positive selection of thymocytes [30]. In this report we found that the development of thymocytes was completely blocked by N17 H-Ras expression, at both high and low expression levels. As discussed above, the difference may due to targeting N17 H-Ras expression into different cells. Lck is first expressed in double negative (DN) thymocytes, whereas MSCV may target N17 H-Ras expression into earlier T lymphocyte precursors and/or lymphoid progenitors. Our results suggest that Ras activation is critical for early thymocyte development.

Similar to the effect of N17 H-Ras in B-lymphocyte development, high expression of N17 H-Ras completely suppressed erythopoiesis, whereas low expression of N17 H-Ras inhibited the expansion of BFU-E, but not CFU-E. A recent study also showed that dominant negative Ras does not reduce CFU-E colony formation [31].

In contrast to the critical role of Ras activation in lymphopoiesis and erythropoiesis, myelopoiesis seemed not to be blocked by expression of N17 H-Ras. It is possible that there are redundant pathways downstream of myeloid growth receptors that regulate proliferation and survival of myeloid cells. It has been shown that both oncogenic Ras and STAT5 can induce myeloproliferative disease [32-34], yet adult hematopoiesis appears normal in STAT5A/B-deficient mice [35]. These two pathways may overlap in promoting myeloid cell proliferation and survival.

As mentioned in the introduction, BCR-ABL with a mutation at the tyrosine-177 residue – a high affinity-binding site for the Grb2 SH2 domain [13,14] – induced a T cell leukemia and lymphoma after a prolonged latent period [15-17]. Consistent with the idea that Ras is a critical downstream target of Y177, N17 H-Ras greatly attenuates BCR/ABL's ability to induce the fatal CML-like MPD. However, unlike the BCR/ABL Y177F phenotype, BCR/ABL-GFP + N17 H-Ras mice developed B, instead of T, lymphoblast leukemia/lymphoma. These results suggest that the Y177 motif in BCR/ABL also regulates signaling pathway(s) other than Ras. Indeed, in addition to recruiting the Ras activator Sos, Grb2 also binds the scaffolding adapter Gab2, which in turn recruits SHP2 and PI3K [18]. It has been shown that the ability of BCR/ABL to confer cytokine-independent growth of primary myeloid cells isolated from Gab2^-/- ^mice in vitro was significantly reduced [18]. These results suggest the latter signaling pathways also play important roles mediating Y177 function in BCR/ABL.

Inhibiting Ras activation by N17 H-Ras has its limitations, as using dominant negative mutants in general. For example, Ras GEFs may have other substrates. Inhibiting Ras GEF, therefore, may affect molecules other than Ras. However, our findings show that the effect of N17 H-Ras in hematopoiesis is lineage, developmental stage, and dosage dependent. This differential role of Ras in the reconstitution and development of different blood lineages and in leukemogenesis warrants further studies.

## Conclusion

In this study we assessed the importance of Ras in BCR/ABL leukemogenesis and the potential toxicity of inhibiting Ras in normal hematopoiesis and found that inhibition of Ras activation by N17 H-Ras suppresses the development of erythrocytes, T and B-lymphocytes, but not myeloid cells. In addition, the presence of N17 H-Ras greatly attenuated BCR/ABL-induced chronic myelogenous leukemia (CML)-like myeloproliferative disease. Most BCR/ABL + N17 H-Ras mice eventually developed B lymphoblastic leukemia/lymphoma. These results suggest that Ras activation is important for the development of lymphoid and erythroid cells but not myeloid cells, and that Ras is a critical target of BCR/ABL in the pathogenesis of CML, but not B-ALL.

## List of abbreviations

CML: Chronic myelogenous leukemia. B-ALL: B-lymphoblastic leukemia. HVR: hypervariable domain. MPD: myeloproliferative disease. GEF: guanine nucleotide exchange factor. SH-2: Src-homology-2. SH-3: Src-homology-3. BM: bone marrow. BMT: bone marrow transplantation. WBC: white blood cell. PBS: phosphate-buffered saline. Epo: erythropoietin. GFP: green fluorescent protein. TCR: T cell receptor. CFU-E: colony-forming unit-erythroid. BFU-E: burst-forming units-erythroid. Neo: neomycin resistant gene. IRES: internal ribosomal entry site. LTR: long terminal repeat. MSCV: murine stem cell virus. N17 H-Ras: A dominant negative mutant of Ras proteins that contains an asparagine at position 17 of H-Ras. PI3k: phosphoinositide-3 kinase.

## Competing interests

The authors declare that they have no competing interests.

## Authors' contributions

RR designed research, analyzed data, and drafted the manuscript. KB participated in the design of the study, performed research, analyzed data, and drafted the manuscript. All authors read and approved the final manuscript.
